# The Triumph of Substance: Decoding the “Functional Infotainment” Model for Sex Education on Douyin

**DOI:** 10.3390/bs15091226

**Published:** 2025-09-09

**Authors:** You Shi, Hao Gao

**Affiliations:** School of Journalism & Communication, Nanjing Normal University, Nanjing 210097, China; 230202073@njnu.edu.cn

**Keywords:** sex education, short video, infotainment, health communication, user engagement

## Abstract

Objective: In the digital age, short-video platforms are key channels for adolescents’ sex education, yet content strategies and their effects remain unclear. This study analyzes Douyin using an integrated source–content–effect framework, identifies infotainment strategies by creator type, and examines their impact on interaction and topic engagement. Methods: Quantitative content analysis of 465 sex-education videos. Content was coded on informational and entertainment value. Four information–entertainment combinations were tested. Engagement outcomes (likes, comments, favorites, shares) were modeled with negative binomial regression; the likelihood that comments were sex-education–related was modeled with logistic regression. Creator type (medical professionals vs. individual creators) entered as a covariate. Results: A functional-infotainment pattern emerged. High information–high entertainment performed best across all interaction metrics. Low information–high entertainment (pure entertainment) performed worst, significantly suppressing deeper engagement and topical discussion. Medical professionals emphasized medicalized, low-risk knowledge; individual creators covered more diverse topics yet likewise avoided sensitive issues. Conclusions: Under algorithmic incentives and cultural norms, Douyin’s sex-education content is not entertainment-first. Dissemination is driven by information-rich content delivered through a functional-infotainment model. Findings refine infotainment theory and offer data-driven guidance: prioritize informational value while pairing it with engaging forms (creators), support high-information content and proactive governance (platforms), and inform education policy.

## 1. Introduction

In recent years, sex education has become increasingly important in global public health. The World Health Organization defines sex education as a fundamental human right, encompassing reproductive health, gender equality, and sexual rights (WHO, 2015). Sex education supports individual physical health and psychological well-being and is also closely linked to social and cultural structures, educational equity, and intergenerational knowledge transmission (Keogh et al., 2020). In particular, timely access to accurate, science-based sex education is critical for adolescents: it helps prevent sexually transmitted diseases, reduces unintended pregnancies, and enhances self-protection capabilities (Chandra-Mouli et al., 2015). In China, sex-related social issues have shown a clear trend toward younger age groups in recent years; among those aged 15–24, the incidence of sexually transmitted diseases continues to rise, and cases of unintended pregnancies and sexual violence among minors are also increasing (Xie et al., 2021; Liu et al., 2023). However, traditional sex education in China has long lacked structured channels, with limited school coverage and a tendency for public discourse to avoid or obscure these topics (Lyu et al., 2020).

Against the backdrop of rapid digital communication, short-video platforms have become important channels for the public to access health information. Among them, Douyin, China’s most influential short-video platform, has reshaped the forms and pathways of health communication through algorithmic recommendations, content decentralization, and strong interactive mechanisms (Kohler & Dietrich, 2021). According to data from the China Internet Network Information Center, more than 30% of Douyin users are between the ages of 18 and 30, a demographic that is among the most underserved groups for sex education (CNNIC, 2024). Compared with traditional text-based media, Douyin’s vivid and easily digestible format gives sex education content greater reach and communicative potential (Hendrickx & Vázquez-Herrero, 2024). Recent evidence also shows how adolescents encounter and use sexuality-education content online in China and comparable settings. In China, a cluster-randomized trial showed that an internet-based sexuality-education program improved adolescents’ sexual knowledge, attitudes, and reported behaviors, indicating that digital delivery can be effective (Hu et al., 2023). On Chinese video platforms, a 2024 study of sexuality-education videos on Bilibili documented quality characteristics together with patterns of audience engagement, suggesting that short-video settings can support both exposure and interaction around educational content (Li et al., 2024). Beyond China, qualitative and survey evidence shows that young people frequently encounter sex- and relationship-related information incidentally in social feeds (e.g., Instagram), and that such exposure can shape what they pay attention to and discuss—even when not actively searching for it (Olsson et al., 2024). At the same time, studies of TikTok highlight mixed information quality and the presence of misinformation, underscoring the value of pairing production-side analysis with reception-side safeguards and measurement (Kirkpatrick & Lawrie, 2024). Complementary evidence from North America and Europe shows that sex-education content is actively produced and consumed on major social platforms: a U.S. content analysis of TikTok sex-education videos catalogued recurring themes and highlighted the platform’s reach to adolescents (Fowler et al., 2022); and a 2024 study in Sex Education analyzed Instagram-based sex-education accounts, detailing how educators structure posts and foster engagement around learning-oriented messages (Sciberras & Tanner, 2024).

Nevertheless, the dissemination of sex education information on Douyin faces multiple challenges. On one hand, sex education, as a highly culturally sensitive topic, is often subject to both platform moderation policies and broader social norms (de Kloet et al., 2019). On the other hand, the algorithmic preference for highly visible and emotionally charged content pushes creators to adapt their styles to maintain communication efficiency, resulting in the “infotainment” of sex education content (Brants & Neijens, 1998; Marinov, 2020). Within this communication ecosystem, medical professionals and institutional accounts, along with ordinary individual creators, together constitute the main content producers. The former are generally regarded as authoritative and professional sources, whereas the latter tend to come from more grassroots creative backgrounds. However, the existing literature still lacks systematic comparisons between these two types of sources in terms of content characteristics, expressive approaches, and audience responses in sex-education-related short videos.

Infotainment refers to the deliberate use of entertainment elements in content whose primary purpose is to convey information. The concept emerged from observations of a shift toward soft news and was first applied in political communication research. It was later extended to other public issues, including health, disaster, and environmental communication (Bernhard & Scharf, 2008; Mellado, 2020; Davis et al., 2020). In the context of sex education, infotainment is not only a way to capture user attention but also a strategic response by creators seeking visibility within platform rules (Tsuriel et al., 2021). This strategy is especially evident on platforms such as Douyin, where algorithmic rankings are driven by user interactions and where content structure and dissemination logic often require creators to balance “watchability” with “tellability” (García-Ortega & García-Avilés, 2023).

While infotainment characterizes supply-side production choices, audience-side mechanisms clarify why these choices matter. A concise reception/mediated-learning perspective suggests that: (a) Entertainment–Education links engaging formats to gains in knowledge and attitudes; (b) Narrative Transportation explains how short, personalized, humorous, story-like formats facilitate processing and reduce counter-arguing; and (c) Social Cognitive Theory accounts for observational learning and self-efficacy as adolescents regularly follow on-camera role models. Together, these perspectives provide a parsimonious rationale for treating information (message substance) and entertainment (presentation cues) as separable, measurable dimensions on short-video feeds, and for expecting their specific combinations to shape what adolescents notice, process, and discuss (Carpena et al., 2024; Green & Appel, 2024; Engel et al., 2024). This integrated framework motivates our itemized measures of both dimensions and the model-based tests reported below.

However, this trend toward infotainment has also raised concerns about possible dilution of content accuracy and authority. Studies note that excessive reliance on entertainment-oriented expression on short-video platforms may weaken the public’s motivation to process and trust serious health information (Newman, 2022). These concerns echo earlier critiques in media research of the soft-news tendency, in which emotional and theatrical expression may reduce informational density and the seriousness of an issue (Reinemann et al., 2012; Zeng et al., 2021). Other scholars have pointed out that, within social media contexts, emotional expression that fails to carry factual information effectively can lead to a performative presentation of health information. There is, however, a more balanced perspective. Some researchers argue that infotainment is not inherently at odds with the goals of health communication; the key is achieving a dynamic balance between entertainment form and informational function (Mellado, 2015). When well designed, infotainment strategies can not only increase user interest but also enhance the accessibility and memorability of information, particularly in highly fluid short-video environments.

At present, systematic studies of infotainment in sex-education content on Douyin remain limited. On the one hand, most research examines informational or entertainment factors in isolation (Peña-Fernández et al., 2022; Vázquez-Herrero et al., 2022), lacking a holistic analysis of their interaction. On the other hand, studies of short-video platforms in the Chinese context often stop at platform mechanisms, policy contexts, or user behaviors, without integrating content characteristics and audience response into multi-dimensional models (Zhang, 2021). In particular, quantitative analyses centered on sex education that assess how creator types and information–entertainment structures jointly influence user engagement, and the generation of public discourse are still lacking.

Moreover, the relationship between interaction effects and communication structure remains to be clarified further. Previous studies often treat interactive behaviors such as likes, comments, favorites, and shares simply as platform feedback (Yao et al., 2024), using interaction volume to measure dissemination effectiveness while overlooking the actual orientation of comment content. In practice, some controversial or purely entertainment-oriented content can also gain high levels of interaction, yet the comment sections may not involve any discussion related to sex education. In such cases, although interaction volume appears substantial, the dissemination effect does not translate into effective diffusion or discussion of sex education topics (Hase et al., 2023). If an information–entertainment structure can significantly increase the likelihood of generating comments relevant to sex education, it indicates that such a structure plays a practical role in promoting discussion of related topics (Waszak et al., 2018). This study examines not only the quantity of interaction behaviors but also uses AI to identify whether comments are related to sex education. This approach extends the evaluation of dissemination effects beyond simple interaction volume to include an assessment of comment orientation, thereby providing a more comprehensive understanding of how information–entertainment structures influence the dissemination of sex education topics.

In summary, a systematic study on the Douyin platform—centered on sex education and using creator type and the information–entertainment structure as dual analytical dimensions—meets practical needs in health communication on Chinese social media, enriches the global theoretical framework on infotainment, and advances public health communication research toward multi-platform, multi-dimensional, multi-source approaches.

To this end, the following research questions are proposed:

RQ1: What thematic types are presented by medical professionals and individual creators in sex-education-related short videos on Douyin?

RQ2: What types of information and entertainment expressions are adopted by medical professionals and individual creators in sex-education-related short videos on Douyin?

RQ3: How do different information–entertainment combinations affect interaction volume in sex-education-related short videos by medical professionals and individual creators on Douyin?

RQ4: How do different information–entertainment combinations affect the likelihood that comments in sex-education-related short videos by medical professionals and individual creators on Douyin address sex education topics?

## 2. Materials and Methods

### 2.1. Research Design

This study employed a quantitative content-analysis approach to systematically examine the dissemination characteristics of sex-education–related content on the Chinese short-video platform Douyin. Accordingly, we adopted quantitative content analysis because RQ3 and RQ4 required statistical modeling to examine how specific message features relate to engagement at scale. This method yields reproducible effect-size estimates with confidence intervals while controlling for confounding variables. In contrast, qualitative or mixed methods are more suitable for context-rich, exploratory analysis and were less appropriate for our large-sample comparative tests. This is a theory-driven quantitative content analysis in the standard sense of the communication-methods literature—coding manifest, item-level features into nominal variables for large-N statistical modeling and effect-size estimation with controls. We searched the platform using keywords such as “sex education” and “sexual health,” drawn from the United Nations Educational, Scientific and Cultural Organization (UNESCO) Comprehensive Technical Guidance on Sexuality Education (UNESCO, 2018) and aligned with our research questions in both meaning and wording. All videos were manually collected through the Douyin app’s search interface under the default “comprehensive ranking.”

During the video screening process, the research team identified user identities according to Douyin’s verification taxonomy. Only accounts bearing a colored “V” explicitly verified in the medical/health category (e.g., physician/hospital/clinic) and disclosing a clinical role or affiliation in the profile were coded as Medical Professionals. Accounts with a colored “V” in any non-medical category (e.g., entertainment, education, enterprise) or without explicit clinical disclosure were coded as Individual Creators. Suspected but unverified medical professionals were treated as ambiguous cases and underwent independent double-coding by two trained researchers using predefined evidentiary cues (e.g., clinical setting or equipment in the video; professional attire plus name badge; hospital/clinic affiliation shown in overlay text or profile; on-screen professional titles). Disagreements were resolved by adjudication, and in the absence of convergent evidence we defaulted to Individual to avoid false positives. In practice, no ambiguous cases arose during data collection.

To ensure the specificity and validity of the sample, we manually filtered out purely commercial advertisements and irrelevant content, ultimately collecting 465 sex-education–related short videos as the final sample. For each video, we extracted likes, shares, favorites, and comments, as well as video duration, publication time, and creator follower count. In addition, we downloaded a total of 12,886 user comments from these videos’ comment sections for analysis.

The publication dates of the collected videos span from June 2024 to February 2025. This period was chosen based on three considerations: (1) to avoid interference from major public health events or social hotspots and reduce data bias; (2) to cover a sufficiently long-time span to generate a representative video sample; and (3) to control for potential seasonal influences on topic preference. During sample collection, we randomly selected content from two groups—medical professionals (including verified doctor accounts) and individual creators. Because the supply of eligible videos varied across months and sources, strict monthly quotas were not enforced; the final distribution reflects platform availability. Each short video was treated as an independent coding unit for subsequent variable measurement and analysis. Moreover, all videos included in the analysis met the criterion of having more than 500 likes to ensure a certain level of dissemination effectiveness.

#### Ethical Considerations and Data Governance

All data analyzed were publicly available on Douyin at the time of collection. We did not interact with platform users, request private information, or attempt to identify individuals beyond public account names. Data collection complied with the platform’s terms of service and relied on manual retrieval via the app’s default “comprehensive ranking” search (no automated scraping or circumvention). Under our institution’s policy, research using publicly available online content without identifiable private information does not constitute human-subjects research; Institutional Review Board (IRB) review was therefore not required. To minimize privacy risks in this sensitive domain, we (i) analyzed interaction counts and video-level features; (ii) used comment text only to generate a binary label (sex-education–related vs. unrelated) based on a prespecified codebook and fixed prompt; (iii) did not retain user identifiers beyond transient processing and avoided verbatim quotations (illustrative examples were paraphrased); and (iv) stored data on encrypted drives with restricted access and time-limited retention for reproducibility.

### 2.2. Themes

Regarding the types of sex education content, we referred to UNESCO’s International Technical Guidance on Sexuality Education (Liu et al., 2023; Li et al., 2024) as well as the International Planned Parenthood Federation (IPPF) framework for Comprehensive Sexuality Education (CSE) (IPPF, 2010). Eight content categories were identified: (1) gender (for example, exploring gender roles and gender bias); (2) reproductive health (for example, prevention of sexually transmitted diseases); (3) contraception and pregnancy; (4) sexual violence and rights (for example, prevention of sexual harassment and consent); (5) sexual pleasure; (6) the human body and development (for example, the anatomy of reproductive organs and physical changes during puberty); (7) sexual minorities (including lesbians, gay men, bisexuals, transgender people, and queer individuals); and (8) relationships (for example, romantic intimacy, trust, and partner communication). Videos that covered multiple themes were coded according to the most prominent theme.

### 2.3. Information

Previous research assessing the quality of video information on social media platforms has not established a unified standard for video quality evaluation. Therefore, the quality checklist used in this study was adapted from multiple sources aligned with our research context, including video quality assessments (Morain & Swarts, 2012) and sex education program evaluations (Li et al., 2024). The checklist consists of six items: (1) relevance (no information that distracts from the purpose); (2) completeness (the video has a clear structure, explicit objectives, and well-defined steps); (3) comprehensibility (the video uses plain language and defines terms when used); (4) accuracy (the information is evidence-based, data are reliable, and sources are cited); (5) usefulness (the video is content-rich and enables viewers to learn effectively); and (6) audiovisual quality (the sound is clear, the volume is appropriate, and the visuals have sufficient lighting and clarity). Each item was coded as absent (0) or present (1) and then summed into an information index (0–6).

These items correspond to widely recognized message-quality facets—journalistic canons (relevance, completeness), usability/plain-language (comprehensibility), evidence transparency (accuracy), practical value (usefulness), and media fidelity (audiovisual). Short-video work on TikTok and BiliBili likewise codes discrete informational elements and links them to quality/engagement outcomes (Chen et al., 2024; Hendrickx & Vázquez-Herrero, 2024). This binary, item-level operationalization minimizes coder discretion, preserves conceptual clarity, and yields analyzable counts and indices appropriate for large-N tests. Video-level coding procedures and inter-rater reliability (Cohen’s κ range 0.734–0.937) are reported in Section 2.6. These six items are formative indicators of information (not reflective latent factors); therefore, binary counts and index summation are appropriate for modeling. Although the information index is a formative composite, we report an exploratory Cronbach’s α of 0.419 for transparency. This modest α is expected because the items target complementary, nonredundant facets rather than a single latent factor. The figure is reported for transparency only and is not used as a reliability criterion.

### 2.4. Entertainment

Based on existing studies on the measurement of infotainment (Ryffel & Wirth, 2020; Jebril et al., 2013; Davis et al., 2020), entertainment characteristics can be operationalized through dimensions such as “narrativity,” “emotional expression,” “personalization,” and “dramatization.” Considering the highly interactive and visually oriented features of short-video platforms, this study further incorporates “interactivity” and “visual impact” as additional dimensions, treating them as important components of entertainment features. In the end, six aspects of entertainment elements in short videos were measured: (1) emotionalization, coded separately for text and visuals and then combined into a binary measure (presence or absence of emotionalization); (2) personalization (whether the video presents a personal story as the main content); (3) dynamic presentation (whether the video includes animations or background music); (4) narrative techniques (whether the video adopts humor, irony, memes, exaggeration, or dramatized storytelling); (5) interactivity (whether the video explicitly encourages viewers to engage, such as by liking, commenting, or sharing); and (6) visual impact (whether the video displays strong visual effects). Each element was coded in binary form (present or absent) and then summed into an entertainment index (0–6).

The selection is theory-driven. Emotionalization captures attention and sharing; personalization supports identification and narrative transportation; dynamic presentation raises message sensation value (MSV) and arousal; narrative techniques provide compact structure that aids encoding; interactivity is a documented production tactic on Instagram/TikTok; and visual impact functions as a format-intensity lever. Cross-platform analyses and Chinese short-video evidence link such production/content features to engagement, supporting their status as formative, countable signals under controls (Vázquez-Herrero et al., 2022; Cheng & Li, 2024; Stephenson & Palmgreen, 2001). Coding workflow and reliability for video features are detailed in Section 2.6. Although the entertainment index is a formative composite, we report an exploratory Cronbach’s α = 0.341 for transparency. This modest α aligns with a formative design in which items tap distinct, complementary facets. The figure is provided for transparency only and is not used as a reliability criterion.

### 2.5. Infotainment

This study adopted the “dual-dimension” measurement strategy for infotainment from previous research (Wirz & Zai, 2025), scoring the informational and entertainment levels of each video as continuous variables. In subsequent analysis, to construct discrete combined variables, we defined scores of 1–3 as “low” and 4–6 as “high,” thereby categorizing the information and entertainment levels into “high” and “low” groups. Accordingly, all videos were classified into four content styles: (1) high-information/high-entertainment (high informational value and high entertainment); (2) high-information/low-entertainment (high informational value but low entertainment); (3) low-information/high-entertainment (high entertainment but low informational value); and (4) low-information/low-entertainment (low informational value and low entertainment). In the regression models, the low-information/low-entertainment group was used as the reference category to examine the effects of different content styles on comment topics and interaction behaviors.

### 2.6. Video Content Coding

Before formal coding began, we discussed each element of the coding scheme with three coders. Then, two coders independently coded 100 randomly selected videos to assess inter-rater reliability. Using Cohen’s kappa, reliability values ranged from 0.734 to 0.937 (*p* < 0.05), which is statistically acceptable and meets the usual requirements for content analysis. The two coders then discussed minor differences and refined the coding standards. After reaching consensus on the coding criteria, the two coders watched all 465 sampled videos and coded them independently. To reduce subjectivity in coding, each coder was assigned video samples from different creator types, ensuring consistency and objectivity in coding.

### 2.7. Comment Content Coding

OpenAI’s Generative Pre-trained Transformer 4 (GPT-4), a large language model, was used to assist with comment-level semantic coding. Hereafter, it is referred to as GPT-4. Following (Wang et al., 2025), we used GPT-4 to analyze comments and determine whether they were related to sex education, and we developed classification rules based on semantic criteria. A comment was classified as sex-education–related if it contained content such as dissemination of sex-education knowledge, contraception methods, sexual knowledge, adolescent education, or HPV vaccine awareness. For example, phrases like “tell it to my daughter,” “schools should teach this,” or “educate from an early age,” as well as references to audiences, dissemination methods, or feedback on understanding, were considered related. Comments consisting only of emojis, interjections, repeated phrases, or only usernames were classified as unrelated. From the full dataset, the research team randomly selected 2000 comments, which were independently coded by two researchers to build the training set. For the dimension of whether the comment was related to sex education, intercoder agreement was high (Cohen’s κ = 0.891, *p* < 0.001), indicating a reliable classification framework. We then used GPT-4 to perform batch semantic identification and automatic classification on the full dataset, creating a binary variable of sex-education–related (1 = related, 0 = unrelated) for subsequent analysis. Following prior work, we used GPT-4 to classify whether comments were sex-education–related; the brief tool-choice justification is provided in Appendix A.

After applying the model to the entire corpus, we randomly sampled approximately 500 comments per class for manual verification to estimate accuracy. The overall accuracy for the sex-education–related label was 96.7% (±0.6%); class-specific accuracies were 94.3% (±1.2%) for related and 97.9% (±0.4%) for unrelated. These values meet common thresholds for academic analyses. Together with the high intercoder agreement in training, these results support using the GPT-4 classifications as the basis for subsequent analyses of user comments on sex-education topics.

### 2.8. Analysis

This study aimed to systematically evaluate how the combination of informational and entertainment elements in videos affects users’ willingness to engage with sex education topics and their actual interactive behaviors, thereby revealing potential mechanisms linking information structure and user responses on short-video platforms. To achieve this, we first conducted Chi-square Tests of Independence to examine differences in performance across content production and user response dimensions: on the one hand, we analyzed whether there were structural differences in the levels of information quality and the frequencies of using six entertainment strategies between different sources (medical professionals vs. individual creators); on the other hand, we further examined whether the comment topic structures triggered by the two source types differed significantly. All Chi-square tests were performed using cross-tabulations, and we reported the χ^2^ statistic, degrees of freedom, and *p*-values to assess independence between variables. These analyses provided both theoretical and statistical foundations for setting up the regression models to test the effects of infotainment combinations on comments and interactions.

For regression modeling, we used logistic regression to analyze the binary variable indicating whether a comment was related to sex education, to evaluate the impact of different infotainment combinations on the likelihood of users expressing sex-education–related opinions. For the four count-type dependent variables (likes, comments, favorites, and shares), we used a negative binomial regression model to determine the strength of the impact of different combinations on user interaction behaviors. Compared with traditional Poisson regression, negative binomial regression is better suited to handle the over dispersed data commonly seen on social media platforms (Hilbe, 2011).

The core explanatory variable was a four-category variable constructed from the information and entertainment scores, including high-information/high-entertainment, high-information/low-entertainment, low-information/high-entertainment, and low-information/low-entertainment, with the low-information/low-entertainment group set as the reference category. All models controlled for three covariates: video duration, time since publication, and the creator’s follower count (log-transformed). Separate models were built for the two source types to test the heterogeneous effects of infotainment combinations across different content creators. All analyses were conducted in SPSS 27, and the results are presented as odds ratios (OR) or incidence rate ratios (IRR), with 95% confidence intervals and significance test indicators.

## 3. Results

### 3.1. Sample Composition and Overall Video Topic Distribution

A total of 465 sex-education-related Douyin short videos were collected in this study, most of which were published by medical professionals (*n* = 341, 73.4%), and a smaller portion came from individual creators (*n* = 124, 26.6%). The total number of comments across these videos’ comment sections was 12,886.

To address Research Question 1 (RQ1), we first analyzed the topic categories of all videos. The results showed that the overall content was dominated by “reproductive health” (38.4%), followed by “contraception and pregnancy” (13.1%) and “sexual pleasure” (12.2%), reflecting the platform’s overall orientation toward medical and popular-science topics.

**Figure 1:**
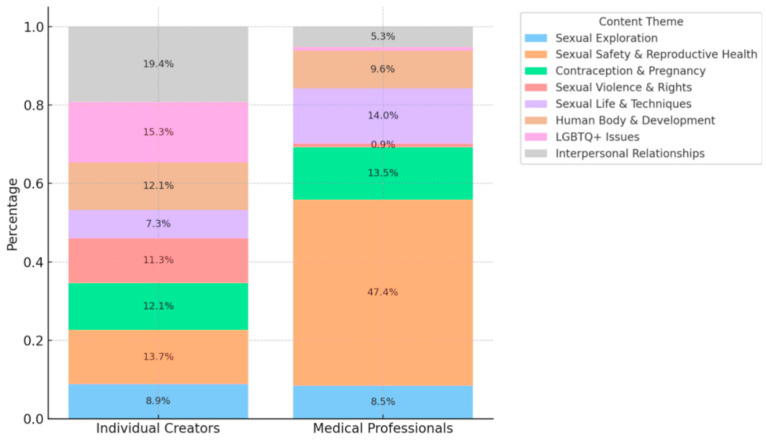
Percentage Distribution of Content Themes Across Creator Types on Douyin.

We further compared differences in video topics across sources. As shown in Figure 1, there was a clear divergence in content presentation between the two source types. Videos from medical professionals were highly concentrated on “reproductive health” (47.4%) and “contraception and pregnancy” (13.5%). In contrast, individual creators presented a wider range of themes, with greater emphasis on social and cultural topics such as “relationships” (19.4%), “sexual minorities” (15.3%), and “sexual violence and rights” (11.3%). The Chi-square test confirmed a statistically significant association between source type and video topic (*χ*^2^(7, *N* = 465) = 118.25, *p* < 0.001), indicating systematic differences in content construction between sources.

### 3.2. Differences in the Use of Informational and Entertainment Strategies

**Figure 2:**
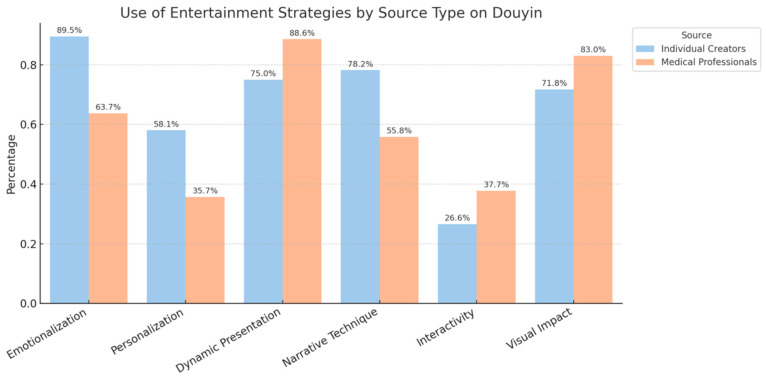
Use of Entertainment Strategies in Sex-Education Videos by Creator Type on Douyin.

**Figure 3:**
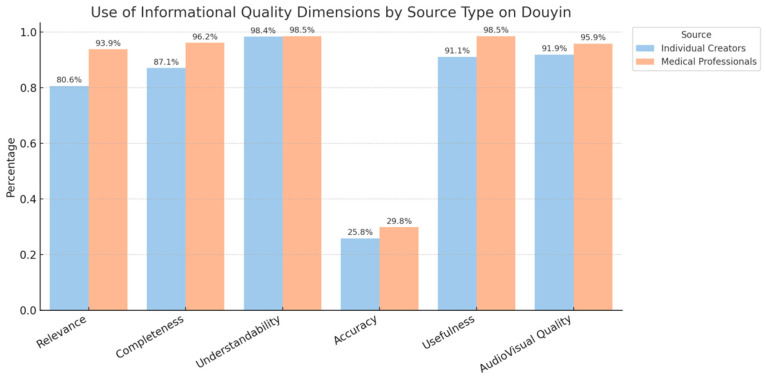
Use of Informational Quality Dimensions in Sex-Education Videos by Creator Type on Douyin.

To address Research Question 2 (RQ2), we systematically coded the video content. As shown in Figure 2, in terms of entertainment strategies, individual creators were more likely to use emotionalization (89.5% vs. 63.7%), personalization (58.1% vs. 35.7%), and narrative techniques (78.2% vs. 55.8%), and these differences were statistically significant (*p* < 0.01). In contrast, medical professionals more frequently used dynamic presentation (88.6% vs. 75.0%, *p* < 0.01). As shown in Figure 3, regarding informational strategies, medical professionals scored significantly higher than individual creators on relevance (93.9% vs. 80.6%), completeness (96.2% vs. 87.1%), and usefulness (98.5% vs. 91.1%) (*p* < 0.01). It is noteworthy that both source types scored very low on accuracy (i.e., whether sources were cited), with scores below 30% and no significant difference between them.

### 3.3. Effects of “Information–Entertainment” Combinations on User Interaction Behaviors

To address Research Question 3 (RQ3), we used negative binomial regression models to examine the effects of four “information–entertainment” combination styles on user interaction behaviors (likes, comments, favorites, and shares). Among the 465 video samples, the “high-information/high-entertainment” combination made up the largest proportion (*n* = 277, 59.4%), followed by “high-information/low-entertainment” (*n* = 168, 36.0%). For model fit, we report pseudo-R^2^ (Nagelkerke) in Table 1 and Table 2. The Nagelkerke values ranged from 0.319 to 0.881, showing that the models fit well for count data and supporting the interpretation of incidence rate ratios (IRRs).

Table 1 and Table 2 present the regression results for videos by medical professionals and individual creators, using “low-information/low-entertainment” as the reference group. Overall, both source types showed a consistent pattern: the “high-information/high-entertainment” combination had the strongest positive effect, followed by “high-information/low-entertainment”. The “low-information/high-entertainment” combination had the weakest effect, and in most cases, it significantly suppressed user interactions. For example, in medical professionals’ videos, the “high-information/high-entertainment” combination increased the number of comments by 64.33 times compared to the reference group (IRR = 64.33, *p* < 0.001). In individual creators’ videos, the same combination increased favorites by 17.55 times (IRR = 17.55, *p* < 0.001). These results show that the integration of high-quality information with entertainment is the strongest driver of user engagement.

For transparency, since the accuracy item in the information instrument was rarely met (2/465), we kept the full sample (*N* = 465) but excluded accuracy from the main analyses and recalculated the information score. We defined “low” as scores of 1–3 and “high” as scores of 4–5, so that “high information” reflects meeting most items (≥4/5 ≈ 80%). Models were also estimated with the original six-item index; both versions led to the same conclusions.

### 3.4. Effects of “Information–Entertainment” Combinations on Comment Content

Finally, to address Research Question 4 (RQ4), logistic regression models were used to evaluate how different combination styles affected whether comment content was related to sex education. As shown in Table 3, the results revealed a clear gradient effect. In both source types, the “high-information/high-entertainment” and “high-information/low-entertainment” combinations significantly increased the likelihood of sex-education-related content appearing in comments. Specifically, in videos by medical professionals, the “high-information/high-entertainment” combination raised the odds of relevant comments to 3.43 times that of the reference group (OR = 3.43, *p* < 0.001). In videos by individual creators, the effect of the “high-information/low-entertainment” combination was also significant (OR = 3.11, *p* < 0.001). In contrast, the “low-information/high-entertainment” combination showed a suppressive effect; in videos by individual creators, this combination significantly reduced the likelihood of relevant comments (OR = 0.38, *p* = 0.010). These findings indicate that purely entertainment-oriented content lacking an informational core not only fails to stimulate deeper user engagement but may even hinder serious discussion of the core topic.

Consistent with 3.3, we retained the full sample (*N* = 465) and used the revised five-item information index (excluding accuracy) with a majority-rule dichotomy (scores ≤ 3 = low; ≥ 4 = high). As a robustness check, models estimated with the original six-item index yielded the same directional patterns and substantively identical conclusions. Regarding model fit, Nagelkerke pseudo-R^2^ is reported in Table 3: 0.133 for medical professionals and 0.229 for individual creators; these values are typical for logistic models with observational data and, together with the significant ORs and non-overlapping 95% CIs, indicate satisfactory overall fit.

## 4. Discussion

This study systematically analyzed the inherent logic and effects of disseminating sex education content—a highly sensitive public health issue—on Douyin, China’s leading short-video platform. By integrating analyses of creator types, information–entertainment strategy combinations, and user interaction behaviors, this research provides new empirical evidence for understanding the health communication ecosystem in the digital era and, more importantly, reveals the dominant role of a Functional Infotainment model: within Douyin’s dual environment of algorithmic operation and cultural context, the most effective dissemination is achieved not through pure entertainment but through the integration of high-quality information with engaging forms. This key finding is significant both theoretically and practically, as it challenges the prevailing belief that entertainment alone guarantees traffic and offers valuable guidance for future health communication practices and platform governance. The following sections elaborate on the main findings from three perspectives: cultural adaptation in content production, the dual effects of platform mechanisms, and functional infotainment and its engagement effects.

### 4.1. Cultural Adaptation in Content Production: Medical Dominance and the Avoidance of Social Issues

Addressing RQ1, an important finding of this study is that sex education content on Douyin shows a clear structural pattern in topic presentation, characterized by medical at the center and social at the margins. A large proportion of videos focus on pure medical popularization with low risk and broad consensus—such as reproductive health, contraception, and the prevention of sexually transmitted diseases—while deeper socio-cultural issues, such as gender identity, sexual minorities, sexual rights, and power structures in intimate relationships, are systematically avoided. We interpret this pattern as a form of strategic avoidance shaped jointly by digital governance and social norms: moderation practices, keyword-sensitive rules, and user reporting interact with cultural expectations about sexuality to narrow what creators perceive as safe frames for expression.

This topic distribution is not accidental; rather, it reflects a process of strategic content adaptation under specific cultural norms, social expectations, and platform moderation pressures. For medical professionals, concentrating on neutral, depoliticized medical knowledge is not only a fulfillment of their professional duty in science communication but also a safe strategy to avoid risks in a complex communication environment. In this context, medical discourse functions not only as the content itself but also as a protective shell for safe expression (Céspedes et al., 2024). For individual creators, topic breadth is wider, yet sensitive issues are approached obliquely—via personal anecdotes, euphemisms, and downplayed labels—to dilute salience and reduce the probability of takedown or social sanction (Chang, 2025). Taken together, visibility on recommendation-driven feeds entails a risk–reward calculus: creators trade educational breadth for perceived safety under algorithmic amplification and rule enforcement, which institutionalizes the medical-centered and socially marginal pattern.

However, a direct consequence of this collective adaptation is a significantly narrowed public understanding of sexual health. Sex education is reduced to technical knowledge, while dimensions such as rights awareness, social responsibility, and interpersonal respect are overlooked. This narrowing is both a governance outcome and a cultural outcome: avoidance of consent, identity, and rights in everyday discourse persists even when biomedical advice circulates widely. Over time, the lack of response to structural social issues and the conditions of marginalized groups has weakened the potential of sex education to foster social understanding and reduce bias (Barriuso-Ortega et al., 2024), while also leaving those who genuinely need support—such as victims of sexual violence and sexual minorities—without necessary visibility or assistance. For sex education to realize its full value in public discourse, platforms, creators, and policymakers need to work together to foster a more diverse, open, and inclusive expressive environment, to achieve a genuine balance between technical safety and social meaning. To preserve pedagogical breadth without compromising safety, platforms should clarify good-faith educational safe harbors and provide transparent enforcement and appeal criteria for sensitive yet necessary topics; creators can pair clinically grounded information with concise social context—clear definitions, consent norms, and referral resources—to retain educational meaning while remaining compliant.

### 4.2. The Dual Effect of Platform Mechanisms: The Tension Between Algorithmic Guidance and Content Governance

Addressing RQ2, Douyin’s platform mechanisms exhibit a typical double-edged sword effect in the dissemination of sex education content. Its algorithmic recommendation and content-governance logic interact in ways that both shape creators’ expression strategies and profoundly influence user interaction patterns. By prioritizing and amplifying interaction signals, the platform algorithm effectively encourages creators to actively explore a path of functional infotainment (Shi & Li, 2024; Kite et al., 2023). The analysis in this study shows that, whether operating as doctors with professional backgrounds or as individual creators, both groups attempt, to varying degrees, to strike a balance between informational professionalism and the appeal of form. Doctors, when conducting popular-science communication, deliberately adopt visual methods such as dynamic demonstrations and metaphorical imagery to make abstract knowledge more intuitive and easier to understand; individual creators, on the other hand, often rely on emotional narratives, personal experiences, and contextualized storytelling to evoke audience resonance. Although these two approaches differ in style, both reflect an active adaptation to and creative use of platform algorithm logic (Bailo et al., 2021). This convergence on visibility-optimizing styles carries explicit ethical responsibilities on commercial feeds: for adolescent-facing content, clarity, accuracy, and age-appropriate framing should take precedence over click incentives, with higher sourcing and disclosure standards for medical accounts.

On the other hand, platform content governance also shows clear delays and limitations in practice. During data collection, about 10% of the sampled videos became unavailable just one day after sampling; many of these were high-interaction videos mainly posted by individual creators. This indicates that platform moderation often intervenes only after content has already achieved widespread dissemination (Gillespie et al., 2020). Such post-hoc governance means that misleading content, topics exploiting gray areas, or content intentionally blurring boundaries can still gain exposure and spread in the short term. This not only affects the overall perception of information quality but also raises new questions about the applicability of traditional gatekeeping theory in the digital age: in a traffic-driven ecosystem, the conventional front-end gatekeeping function is weakened, and the platform more often acts as a corrective agent after the fact (Goldstein et al., 2023). This mechanism allows content creators, in the pursuit of traffic, to test boundaries and even exploit moderation delays to gain attention, thereby introducing potential governance risks. Ethically, post-hoc removals do not reverse prior exposure; platforms and creators therefore share a duty to mitigate foreseeable harm through pre-publication verification, in-caption source citations and timestamps, prompt corrections, and to avoid fear appeals or provocative thumbnails unless paired with concrete guidance (Pennycook et al., 2021).

Overall, Douyin has developed a content ecosystem in health communication that centers on information while using entertainment as a supplement, successfully facilitating the emergence of a large volume of high-quality popular science content. However, the sustainability of this model requires caution. On the one hand, the platform algorithm prioritizes and amplifies interaction signals, prompting creators to continuously innovate in expressive form to attract attention; yet the findings of this study show that relying solely on emotional and dramatized entertainment elements does not lead to high interaction. In contrast, content that combines rich informational value with moderate entertainment features truly achieves broad dissemination. On the other hand, delayed content governance allows such practices to be excessively amplified in the short term, leading to risks of dissemination distortion and the spread of health misinformation (Chou et al., 2020). Practically, platform-level guardrails (good-faith education safe harbors with transparent enforcement and appeals, educator verification labels with pre-clearable templates for high-risk themes, and risk-tier review that temporarily throttles unverifiable claims) together with creator-side protocols (source citation, age-appropriate disclaimers, explicit correction/update procedures) can align the observed high-information/high-entertainment pattern with ethical safeguards and reconcile commercial reach with educational integrity. Survey evidence from young women on TikTok underscores concurrent exposure to health misinformation alongside routine use for learning, which strengthens the case for pre-publication verification and transparent corrections on commercial platforms (Kirkpatrick & Lawrie, 2024).

### 4.3. The Dominance of Functional Infotainment: The Informational Core as the Key Driver of User Engagement

In relation to RQ3 (interaction volume) and RQ4 (topic-relevant commenting), the most central finding of this study is that, in the context of sex education, entertainment elements alone do not guarantee dissemination effectiveness; their impact relies heavily on the strength of the informational content. Regression results show that the high-information/high-entertainment combination performed best across all interaction metrics—likes, comments, favorites, and shares—and increased the likelihood that comment threads remained focused on sex-education topics. This pattern affirms the effectiveness of functional infotainment, which meets both cognitive and entertainment needs and most effectively motivates users to participate and share. The synergy between information and entertainment aligns closely with the integrated model of infotainment (Green & Appel, 2024) and the dual-processing mechanism of emotion and cognition in social media (Winkler et al., 2023).

In sharp contrast, the low-information/high-entertainment combination displayed significant negative effects across almost all interaction metrics. This result challenges the entertainment-first dissemination strategy and reveals the critical boundary of infotainment in health communication. When entertainment forms are detached from valuable informational cores—especially on serious and sensitive topics such as sex education—users are not only unlikely to be attracted but may also develop cognitive disengagement and emotional aversion due to the content’s superficiality and flippancy, thereby reducing their willingness to interact. Such negative effects may stem from a mismatch between user expectations and what is provided: when users seek knowledge or solutions but encounter only sensory stimulation or dramatized performances lacking substance, they perceive their informational needs as disregarded, leading to reduced trust in both the creators and their content (Dai & Wang, 2023).

Therefore, this finding offers concrete guidance for platform governance and health communication. Although entertainment can capture attention (Moyer-Gusé, 2008), our results show that entertainment alone neither sustains interaction nor avoids resistance (Cinelli et al., 2021). Many health creators have long treated entertainment as the traffic lever (Kubler, 2023), yet communicative power depends chiefly on information quality and density—especially when information and entertainment work in concert. Creators should therefore prioritize informational value, maintain professional rigor, and integrate entertainment judiciously to support durable engagement. Translating RQ3/RQ4 into intervention design, program owners (educators/public-health agencies) can adopt an information-first template with restrained narrative under standard operating procedures for verification, in-caption sourcing/timestamps, and correction/update protocols, with platforms and creators as delivery partners.

For adolescents, the same evidence supports simple classroom routines: (1) a 2 × 2 grid to sort real posts by information and entertainment; (2) a short pre-share accuracy check—confirm the in-caption source and timestamp and write a one-sentence summary. These routines can be embedded in CSE/health modules and aligned with digital-literacy standards (Vuorikari et al., 2022). Evaluation should target reception outcomes (completion rate, comment topicality, share-through) rather than views alone. Schools can deliver via teacher professional development and age-appropriate scaffolding; public-health agencies can supply vetted content and referral resources; platforms can provide an educational safe-harbor tag to reduce takedown risk for age-appropriate, evidence-based content. Equity safeguards should include accessibility features and culturally sensitive examples to support diverse learners.

## 5. Limitations

First, the information and entertainment indices are formative composites (Methods 2.3–2.4). Their exploratory Cronbach’s alpha values (information 0.419; entertainment 0.341) are modest because items tap complementary facets rather than a single trait; this does not imply poor measurement in a formative design, but random item noise can attenuate observed relationships—so significant effects are conservative lower bounds, and some non-significant results may reflect signal masked by noise. Second, AI-assisted comment labeling can misread sarcasm, evolving slang, code-switching, or emoji-only replies, which would further dilute associations involving comment outcomes. Third, as an observational study, estimates describe associations under observed controls (duration, recency, follower count) and should not be over-interpreted as causal. Finally, external validity is bounded by the platform and period studied (Douyin, China, 2024–2025); generalization beyond this context warrants caution and replication.

## 6. Conclusions

Theoretically, we propose a functional-infotainment model that emphasizes an information-rich core. We also explain how creators adapt their content strategically based on cultural norms and platform governance. Empirically, our analysis of 465 Douyin sex-education videos reveals that the high-information/high-entertainment style performs best in terms of likes, comments, favorites, and shares. It also generates more topic-relevant comments. On the other hand, low-information/high-entertainment performs poorly, with topics clustering around low-controversy medical content. Practically, we recommend a production template that prioritizes information, with a balanced narrative. The basic steps include pre-publication verification, in-caption source and date stamps, and prompt corrections/updates. These should be implemented jointly by platforms and creators.

## Figures and Tables

**Table 1:** Effects of Information–Entertainment Combinations on Interaction Volume in Videos by Medical Professionals (Negative Binomial Regression Results).

Source	Dependent Variable	Style	IRR	95% CI	*p*	Significance	Nagelkerke R^2^
Medical Professional	Likes	Low Information-High Entertainment	0.77	[0.47, 1.27]	0.305		0.433
Medical Professional	Likes	High Information-Low Entertainment	4.63	[3.85, 5.57]	<0.001	***	0.433
Medical Professional	Likes	High Information-High Entertainment	18.34	[15.24, 22.07]	<0.001	***	0.433
Medical Professional	Comments	Low Information-High Entertainment	0.24	[0.14, 0.39]	<0.001	***	0.881
Medical Professional	Comments	High Information-Low Entertainment	16.9	[14.04, 20.35]	<0.001	***	0.881
Medical Professional	Comments	High Information-High Entertainment	64.33	[53.41, 77.47]	<0.001	***	0.881
Medical Professional	Favorites	Low Information-High Entertainment	0.45	[0.27, 0.74]	0.002	**	0.48
Medical Professional	Favorites	High Information-Low Entertainment	1.94	[1.61, 2.33]	<0.001	***	0.48
Medical Professional	Favorites	High Information-High Entertainment	11.49	[9.55, 13.82]	<0.001	***	0.48
Medical Professional	Shares	Low Information-High Entertainment	0.29	[0.17, 0.47]	<0.001	***	0.319
Medical Professional	Shares	High Information-Low Entertainment	8.92	[7.42, 10.73]	<0.001	***	0.319
Medical Professional	Shares	High Information-High Entertainment	22.35	[18.58, 26.90]	<0.001	***	0.319

IRR = incidence rate ratio; CI = confidence interval; *** *p* < 0.001, ** *p* < 0.01. Pseudo-R^2^ (Nagelkerke) reported.

**Table 2:** Effects of Information–Entertainment Combinations on Interaction Volume in Videos by Individual Creators (Negative Binomial Regression Results).

Source	Dependent Variable	Style	IRR	95% CI	*p*	Significance	Nagelkerke R^2^
Individual Creator	Likes	Low Information-High Entertainment	0.29	[0.24, 0.36]	<0.001	***	0.48
Individual Creator	Likes	High Information-Low Entertainment	3.48	[3.1, 3.91]	<0.001	***	0.48
Individual Creator	Likes	High Information-High Entertainment	11.13	[9.98, 12.41]	<0.001	***	0.48
Individual Creator	Comments	Low Information-High Entertainment	0.18	[0.15, 0.22]	<0.001	***	0.447
Individual Creator	Comments	High Information-Low Entertainment	5.13	[4.57, 5.75]	<0.001	***	0.447
Individual Creator	Comments	High Information-High Entertainment	10.47	[9.39, 11.67]	<0.001	***	0.447
Individual Creator	Favorites	Low Information-High Entertainment	0.26	[0.21, 0.31]	<0.001	***	0.441
Individual Creator	Favorites	High Information-Low Entertainment	11.7	[10.43, 13.13]	<0.001	***	0.441
Individual Creator	Favorites	High Information-High Entertainment	17.55	[15.74, 19.57]	<0.001	***	0.441
Individual Creator	Shares	Low Information-High Entertainment	0.04	[0.03, 0.05]	<0.001	***	0.459
Individual Creator	Shares	High Information-Low Entertainment	7.85	[7.0, 8.81]	<0.001	***	0.459
Individual Creator	Shares	High Information-High Entertainment	9.59	[8.6, 10.7]	<0.001	***	0.459

IRR = incidence rate ratio; CI = confidence interval; *** *p* < 0.001. Pseudo-R^2^ (Nagelkerke) reported.

**Table 3:** Logistic Regression Results on the Effects of Information–Entertainment Combinations on Whether Comment Content Is Related to Sex Education.

Source	Group	OR	95% CI	*p*	Significance	Nagelkerke R^2^
Medical Professional	Low Information-High Entertainment	0.55	[0.07, 4.53]	0.582		0.133
Medical Professional	High Information-Low Entertainment	2.93	[1.62, 5.31]	<0.001	***	0.133
Medical Professional	High Information-High Entertainment	3.43	[1.90, 6.20]	<0.001	***	0.133
Individual Creator	Low Information-High Entertainment	0.38	[0.18, 0.79]	0.010	**	0.229
Individual Creator	High Information-Low Entertainment	3.11	[2.28, 4.25]	<0.001	***	0.229
Individual Creator	High Information-High Entertainment	2.10	[1.54, 2.86]	<0.001	***	0.229

OR = odds ratio; CI = confidence interval; *** *p* < 0.001, ** *p* < 0.01. Pseudo-R^2^ (Nagelkerke) reported.

## Data Availability

The data presented in this study are available on request.

## Appendix A

Given the scale and colloquiality of our corpus (12,886 comments), an AI-assisted coding workflow was implemented with explicit attention to the limitations of generative models and the complementary role of traditional software. NVivo supports manual and dictionary/rule-based coding, but at this scale—and with slang, emojis, code-switching, irony, and extremely short posts—such pipelines remain labor-intensive, are brittle to evolving vernacular, and require continual retuning; SPSS is a statistical environment that does not provide context-aware text classification without substantial bespoke preprocessing and supervised labeling. At the same time, using a generative model as a semantic analyzer carries important caveats in colloquial social-media text: opacity and prompt sensitivity, nondeterminism and version drift, potential misreadings of pragmatics (sarcasm, implicitness, regional slang, emoji semantics, intertextual references), subgroup/domain biases, and calibration drift as platform language changes. Risks were mitigated by fixing model and decoding settings, prespecifying a concise codebook and single prompt, logging model IDs and the full prompt for reproducibility, applying stratified double-coding with adjudication, and auditing known hard cases (e.g., sarcasm, emoji-only/heavy) to refine decision rules. In this configuration, GPT-4 provides scalable, context-sensitive classification, whereas traditional tools remain in our workflow for data management and statistical inference. Accordingly, generative AI complements—rather than replaces—conventional software, and the prompt, rules, and validation are documented for transparency and replicability.
